# High Sensitivity Singlet Oxygen Luminescence Sensor Using Computational Spectroscopy and Solid-State Detector

**DOI:** 10.3390/diagnostics13223431

**Published:** 2023-11-12

**Authors:** Tiffany C. Yu, Steve J. Davis, Mark T. Scimone, John Grimble, Gopi Maguluri, Sanjay Anand, Cheng-En Cheng, Edward Maytin, Xu Cao, Brian W. Pogue, Youbo Zhao

**Affiliations:** 1 Physical Sciences Inc., Andover, MA 01810, USA; tyu@psicorp.com (T.C.Y.);; 2 Cleveland Clinic, Cleveland, OH 44195, USA; 3 Thayer School of Engineering at Dartmouth, Hanover, NH 03755, USA

**Keywords:** singlet oxygen, photodynamic therapy, UV skin irradiation, skin cancer, luminescence spectroscopy

## Abstract

This paper presents a technique for high sensitivity measurement of singlet oxygen luminescence generated during photodynamic therapy (PDT) and ultraviolet (UV) irradiation on skin. The high measurement sensitivity is achieved by using a computational spectroscopy (CS) approach that provides improved photon detection efficiency compared to spectral filtering methodology. A solid-state InGaAs photodiode is used as the CS detector, which significantly reduces system cost and improves robustness compared to photomultiplier tubes. The spectral resolution enables high-accuracy determination and subtraction of photosensitizer fluorescence baseline without the need for time-gating. This allows for high sensitivity detection of singlet oxygen luminescence emission generated by continuous wave light sources, such as solar simulator sources and those commonly used in PDT clinics. The value of the technology is demonstrated during in vivo and ex vivo experiments that show the correlation of measured singlet oxygen with PDT treatment efficacy and the illumination intensity on the skin. These results demonstrate the potential use of the technology as a dosimeter to guide PDT treatment and as an analytical tool supporting the development of improved sunscreen products for skin cancer prevention.

## 1. Introduction

Singlet oxygen (^1^O_2_) is a highly energetic reactive oxygen species (ROS) that plays significant roles in many biological processes [1,2]. The excited and highly reactive species may interact with most biomolecules, including lipids, proteins, and DNA/RNA [3]. Exposure to singlet oxygen thus causes a variety of impairments to biological systems and processes [4]. Singlet oxygen is often produced during the type II photosensitizing reactions in the presence of light and photosensitizing molecules or photosensitizers (PS) [5,6]. Singlet oxygen generation in skin during ultraviolet (UV) irradiation is a major cause of skin damage and aging [7,8,9]. There is evidence that singlet oxygen also contributes to DNA damage and thus skin carcinogenesis [10,11,12,13,14,15,16,17]. The use of sunscreens is recommended by dermatologists to block solar UV irradiation and thus prevent skin damage and cancer [18,19]. In photodynamic therapy (PDT), a clinically proven cancer treatment option, singlet oxygen is the primary reactive species that kills tumor cells [20,21,22,23]. Numerous studies have shown that singlet oxygen production is strongly correlated with PDT treatment outcome [23,24,25,26,27,28,29,30,31]. The production rate of singlet oxygen is a key concern in the development of PS drugs and clinical PDT procedures. Real-time measurement of singlet oxygen thus holds great value in the investigations of these biological processes in which singlet oxygen plays a crucial role [24,30,31,32,33,34,35]. Singlet oxygen dosimetry is critically needed in PDT clinics to provide real-time dosing guidance and feedback to optimize patient outcomes. It also provides important data to guide the development of both new generation PSs for PDT and more effective sunscreen products for improved skin protection.

The development of singlet oxygen detection systems has been extensively explored, and many methods have been demonstrated [33,34,35,36,37]. Among them, optical detection has the advantages of noninvasiveness and real-time measurements. Optical detection of singlet oxygen may be implemented using fluorescent probes or based on direct luminescence measurements. Fluorescent probes provide high sensitivity and specificity and are frequently used in biological research [37,38]. However, they are not suited for in vivo studies due to the requirement for exogenous fluorophore administration. There have been many efforts in developing singlet oxygen sensors targeted for in vivo and clinical use, spurred by the significant need for PDT guidance. It has been shown that singlet oxygen may be quantified by PS fluorescence dynamics [24,26,32]. Strong correlations have been demonstrated between treatment outcome and PS fluorescence bleaching rate in several clinical trials [39,40]. However, this is an indirect measurement, and the calibration between the measured and true singlet oxygen value is complicated, varying with PS properties [34,41]. Alternatively, several PDT studies have demonstrated an approach to quantify singlet oxygen based on assessments of light dose, PS dose, and tissue oxygen concentration, the three parameters contributing to singlet oxygen generation [24,33]. When assisted by macroscopic modeling of singlet oxygen yield, this approach has been proven to be an effective means to predict PDT outcome during in vivo tumor treatment experiments [42,43,44]. However, real-time and accurate measurements of all three parameters under clinical settings are not technically trivial.

Singlet oxygen emits near infrared (NIR) luminescence centered at 1270 nm, enabling direct optical measurement [45,46]. However, optical detection of singlet oxygen luminescence is highly challenging, due to the ultraweak signal level and the presence of strong PS fluorescence background at overlapping wavelengths. Because of the low emission probability (e.g., 10^−8^) and the short quenching lifetime (e.g., <1 µs in tissue environments), singlet oxygen luminescence signal is extremely weak [26,47]. Moreover, this emission is often overwhelmed by PS fluorescence that extends to the 1270 nm wavelength region. To extract the singlet oxygen luminescence signal, a detection system with sufficient spectral resolution and high sensitivity is required. These spectral measurement capabilities are needed to resolve the singlet oxygen luminescence peak from the featureless PS spectral baseline or background [30,31]. In addition, high sensitivity is a requirement for the measurement of weak luminescence signal. So far, the best NIR detectors are photomultiplier tubes (PMTs) due to their large, essentially noise-free amplification. The fast response time of PMTs also allows for time-gated measurements to achieve temporal discrimination of the singlet oxygen luminescence from the longer-lived PS fluorescence background [29,48,49,50]. To achieve spectral resolution, a number of bandpass filters are often the best choices. Despite the high transmittance of the bandpass filters, the signal detection efficiency of this approach is intrinsically low. This is because each filter only transmits a narrow wavelength band, discarding (blocking) all other out-of-band wavelength components. In this case, the signal detection efficiency is described as δλΔΛ, where δλ is the bandwidth of the filters, and ΔΛ is the measured spectral range. In our previous efforts, we developed a high-throughput spectrometer using a thermoelectrically cooled 2D array detector that provided high photon detection efficiency to mitigate this issue [30]. However, the spectrometer has a high cost, primarily driven by the camera, which costs more than USD 100,000.

In this paper, we present a new method for high sensitivity measurement of singlet oxygen emission using a computational spectroscopy (CS) technique. The CS approach allows both high spectral resolution and high signal throughput while significantly reducing cost by using a spatial-light modulator and a single-point solid-state detector. This approach enables non-invasive detection of singlet oxygen luminescence with sufficient sensitivity and spectral resolution, enabling real-time measurement of singlet oxygen produced during UV irradiation of skin and during PDT treatment. In vivo PDT and ex vivo skin irradiation experiments are reported to demonstrate the value of this technology in supporting UV skin irradiation investigations and PDT treatment dose optimization.

## 2. Materials and Methods

**Figure 1:**
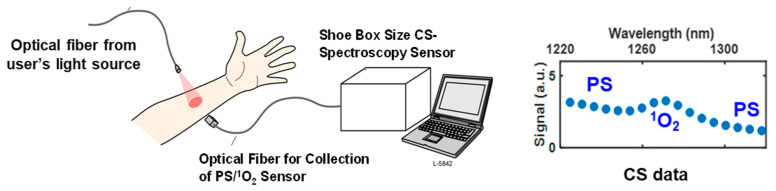
Conceptual application of the fiber-coupled, computational spectroscopy (CS) singlet (^1^O_2_) luminescence sensor.

The concept of the CS singlet oxygen detection system is illustrated in Figure 1. A shoebox-sized CS-based spectrometer is used to detect singlet oxygen luminescence generated in tissue during PDT or skin UV irradiation. The device has a flexible fiber bundle and a high efficiency light collector that is placed at a distance of >20 mm from the irradiated area. The dosimeter does not come in contact with the skin and thus does not interfere with the PDT treatment or UV exposure studies. The detection system does not require any additional light sources, but rather collects singlet oxygen luminescence signal excited by the light sources used for PDT treatment or commercial UV solar stimulator sources for skin studies.

**Figure 2:**
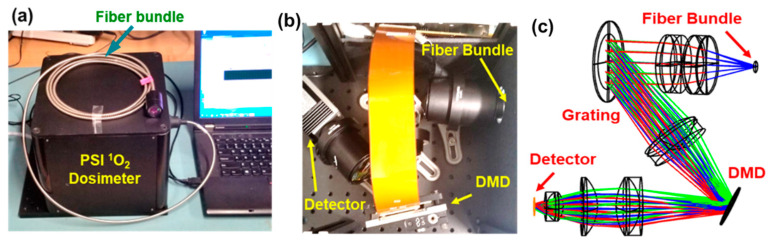
Photographs (**a**,**b**) and optical layout (**c**) of a prototype computational spectroscopy (CS) singlet oxygen (^1^O_2_) spectrometer using a digital micromirror device (DMD).

The CS spectrometer sensor prototype is shown in Figure 2. The CS spectrometer consists of a collimation lens group, a transmission grating, an imaging lens group, an NIR digital micromirror device (DMD, DLP650LNIR, TI), a focusing lens group, and a thermoelectrically cooled, single-point InGaAs photodiode (59141, Edmund Optics, Barrington, NJ, USA). The prototype has dimensions of 30.5 cm (L) × 13.5 cm (W) × 45.7 cm (H). A gooseneck conduit-protected fiber bundle is used to collect the luminescence signal of both PS and ^1^O_2_. The fiber bundle has a circular proximal end (3 mm in diameter) to collect luminescence signal and a linear distal end (0.9 mm × 11 mm) that is coupled to the CS spectrometer optical assembly.

The optical ray trace design of the spectrometer is shown in Figure 2c. The instrument uses the DMD as a spatial light modulator at the focal plane of the CS spectrometer. The DMD selects combinations of different wavelengths that are detected and recorded by the InGaAs detector. The acquisition of the detector and activation of the DMD is synchronized. After a series of acquisitions, each corresponding to a unique combination of wavelengths, the spectrum is calculated based on a Hadamard transform, as demonstrated by DeVerse et al. in [51]. This approach has the advantage of high signal detection efficiency and thus high signal-to-noise ratio (SNR) due to the fact that each acquisition simultaneously measures multiple wavelength components, in contrast to one narrow wavelength region in numerous sequential measurements using the spectral filter approach. This is equivalent to the Fellgett advantage in Fourier transform infrared spectroscopy [52].

The design of the CS spectrometer is optimized for high throughput and signal collection. The optical throughput or f/# of the spectrometer is 1.3. The transmittance of the entire spectrometer setup is ~41%, which was measured using a laser diode with an emission wavelength of 1310 nm. Due to the intrinsic tradeoff between light throughput and spectral resolution in spectrometer design, we intentionally sacrificed the resolution to maximize throughput. The measured spectral resolution was ~14 nm, as a compromise of the large slit width of the fiber bundle and the optical aberration introduced by the high numeric aperture (high throughput) optics. The spectral resolution may be improved by using more optical elements to correct aberrations and using a narrower slit (i.e., a fiber bundle with fewer fibers). However, all these changes will inevitably lead to reduced light-throughput and photon-collection efficiency, which is the most critical consideration for detecting biologically weak singlet oxygen luminescence. Our data, shown later, demonstrate that the spectral resolution of the system is sufficient to distinguish singlet oxygen emission from PS fluorescence background.

**Figure 3:**
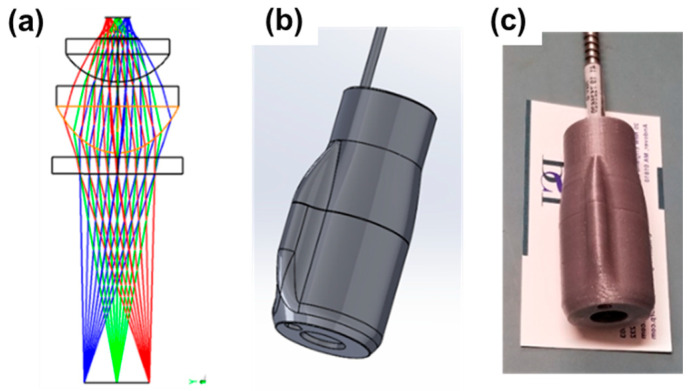
(**a**) Zemax optical design for the fiber light collector utilizing an aspheric lens and a plano-convex lens, Solidworks mechanical design CAD, (**b**) and 3D printed light collector assembly (**c**) (typically sized business card for scale).

To optimize the light collection efficiency, a fiber light collector was designed and fabricated using a high numerical aperture aspheric lens (f = 12 mm, ACL1512U, Thorlabs, Newton, NJ, USA) and a plano-convex lens (f = 15 mm, LA1540C, Thorlabs). The Zemax optical design is shown in Figure 3a. A 3D printed assembly was developed to mount the lenses and the fiber (Figure 3b,c). A miniature visible wavelength camera (XDT311M5, Misumi, New Taipei City, Taiwan) was also incorporated into the 3D printed light collector as an add-on feature. This camera guided the alignment of the fiber collector to the irradiated skin area and collected PS fluorescence in the visible wavelength range (450 nm to 750 nm).

For the in vivo PDT and skin UV irradiation experiments reported, all the procedures followed IACUC protocols approved by the Cleveland Clinic and Dartmouth College, respectively.

### 2.1. In Vivo Actinic Keratoses (AK) Models

For PDT experiments, a cohort of six mice, including two controls and four treated mice, were used in this study. The mice first developed actinic keratoses (AK), pre-SCC lesions on the skin, with repeated (three times a week) UVB irradiation up to 20 weeks. Prior to PDT treatment, the AK lesions were topically treated with 20% ALA dissolved in PBS containing 5% EDTA and 2% DMSO. Following application, the mice were incubated for 1 h in the dark to prevent ambient light from reacting with the ALA. The 1 h incubation time was used to allow the ALA to accumulate at the AK lesions, and the mice were kept in the dark to prevent unwanted ALA activation by ambient light. The mice were then treated using 405 nm light from an LED, using light doses of ~10 J/cm^2^. Tumor sizes were recorded weekly for two weeks following treatment, using a caliper to measure the tumor volume, until humane endpoints were reached according to the approved IACUC protocol. The procedure for the control group that did not receive any ALA application or light exposure was identical to the treatment group.

### 2.2. Skin UV Irradiation

For the UV irradiation experiments, a spectral filtered UV lamp was used as the light source. During the in vivo experiments, a cohort of eight mice was kept anesthetized in the surgical cradle using isoflurane (3% for induction, 1–3% for procedure) with an oxygen flow rate of 1–2 L/min. A toe pinch was used to confirm that complete anesthesia was present, and mice were closely monitored for depth of anesthesia throughout the experiments.

### 2.3. Statistcal Analysis

The measured ^1^O_2_ and PS values were analyzed and tested for correlation with both tumor reduction values and UV powers, respectively, in the PDT and UV irradiation studies. Linear fits were used to determine the degree of correlation. R squared values were determined using a linear regression model which was used to indicate the statistical significance of the correlation. Error bars were determined with standard deviation.

## 3. Results and Discussion

**Figure 4:**
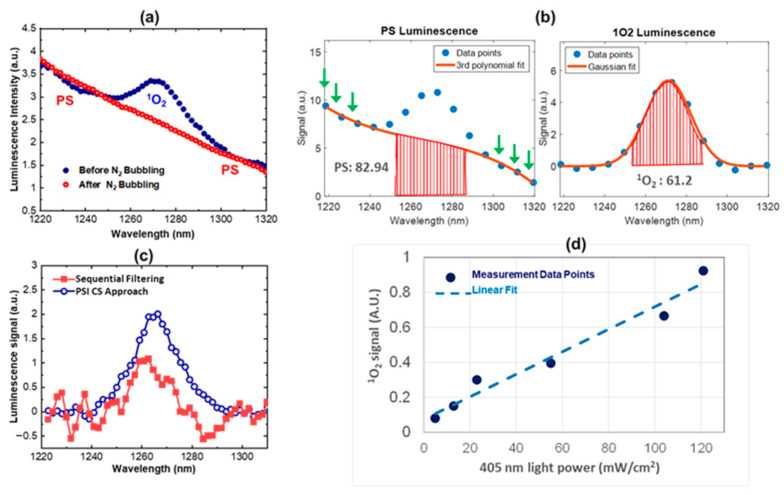
System characterization using PPIX phantom solutions. (**a**) PS/^1^O_2_ spectra of PPIX solution measured before and after bubbling of nitrogen gas; (**b**) raw spectra (**left**) and baseline subtracted spectra; green arrows indicate points to which the baseline is fitted to determine the PS spectral baseline; (**c**) PS/^1^O_2_ spectra measured by the CS approach and traditional sequential spectral filtering approach; (**d**) measured luminescence at different light intensity levels.

The prototype instrument was first characterized using PS solutions. Representative experimental data are shown in Figure 4. In Figure 4a, a (Protoporphyrin IX) PPIX PS phantom was used as the sample, and a 405 nm LED source was used to excite the solution. The ^1^O_2_ peak (centered at 1270 nm) disappeared when nitrogen gas was bubbled through the solution, replacing the oxygen in the solution that reacts with PPIX PS to produce ^1^O_2_. This experiment confirmed that the peak at 1270 nm measured by the CS-based optical detection system is ^1^O_2_ luminescence. In this experiment, 64 spectral data points were used for spectral measurements, which required the same number of DMD frames and signal acquisitions. To optimize the SNR, the DMD frame rate and the detector acquisition were set to be 10 Hz. Therefore, the acquisition time for each CS spectral measurement was approximately 6.4 s.

Figure 4b shows the data processing procedures developed to define the PS baseline and the ^1^O_2_ luminescence. Specifically, a third order polynomial model was fit to the spectral regions out-of-band of ^1^O_2_ luminescence spectrum (covering 1220–1235 nm and 1305–1320 nm). The third order polynomial fit model was empirically determined, providing a method of defining the PS signal and discriminating it from the ^1^O_2_ luminescence signal. The PS baseline was subtracted from the raw data signal and the remaining spectral shape was fit to a Gaussian model without predetermined parameters. The ^1^O_2_ value was then quantified by integrating the Gaussian fit within the wavelength range of 1260 nm to 1280 nm. It should be noted that this determination is a relative number, and the calibration of this measurement with respect to the absolute ^1^O_2_ quantity may be determined using a calibrated black body irradiance source in the future.

Figure 4c shows the ^1^O_2_ luminescence spectra that were measured using the CS spectrometer and the wavelength scanning (spectral filtering) approach. The wavelength scanning approach was achieved by using the DMD to select one wavelength each time. The CS approach has a significantly improved SNR, attributed to the improved light collection efficiency that measures multiple wavelengths simultaneously, in contrast to one wavelength at a time sequentially in the typical wavelength scanning approach. As discussed in [51], the SNR enhancement is given by N2, where N is the number of spectral data points. In this case, the anticipated SNR improvement is 5.7×, which is consistent with the measurements.

Figure 4d shows the measured ^1^O_2_ luminescence at different excitation intensity levels, which has a linear correlation with a goodness of fit of 0.92. These data validate our developed algorithms for PS baseline determination/subtraction and thus singlet oxygen quantification.

The value of the technology in guiding PDT dose management was demonstrated during in vivo PDT experiments. A total of eight mice with squamous cell carcinoma (SCC) lesions on their skin were treated using a 405 nm LED source. The PS fluorescence and ^1^O_2_ luminescence spectra were collected by the CS spectrometer during the treatment. The tumor lesions were measured one week and two weeks post-treatment to assess tumor reduction rate, which is used as the indicator of treatment efficacy.

**Figure 5:**
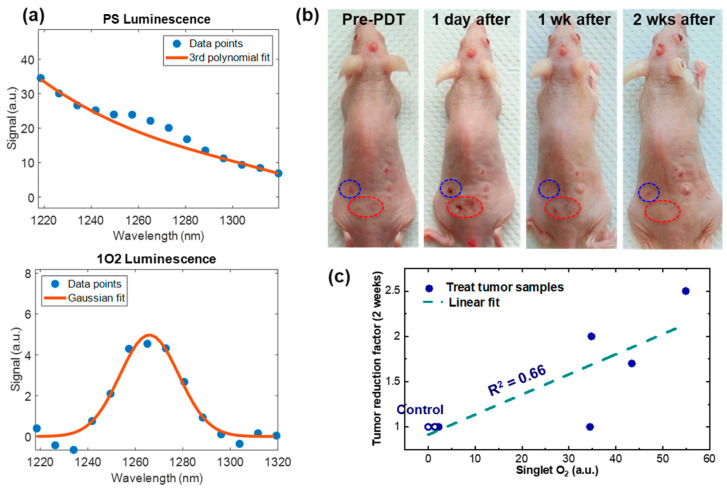
^1^O_2_ measurements during in vivo ALA PDT treatment. (**a**) Recorded spectra for PS (**top**) and ^1^O_2_ (**bottom**) for an SCC model mouse (shown in **b**); (**b**) photographs of the mouse showing tumor regression in PDT treated areas (circles indicate separately treated tumors) at days 1, 7, and 14 following treatment; (**c**) correlation of tumor regression as a function of ^1^O_2_ measured during treatment. The circled data points correspond to the tumor lesions in (**b**).

Figure 5a shows example measurements of PS and ^1^O_2_ from a living mouse during the PDT treatment. High SNR measurements of ^1^O_2_ were demonstrated by the high-fidelity Gaussian fit (fit goodness R^2^ = 0.98) to the measurement data points in the ^1^O_2_ spectrum (lower panel of Figure 5a). This CS spectrometer was calibrated against a blackbody emitter (SR-2-33, CI Systems, Carrollton, TX, USA). To the best of our knowledge, the spectra in Figure 5a are the first high-resolution PS and ^1^O_2_ spectra measured from a living mouse during ALA PDT treatment of skin cancer. Photographs of the tumors pre- and post-treatment are shown in Figure 5b. Visual inspection indicates that in the treated areas, i.e., within the circled areas, tumor shrinkage is clearly shown over the two-week period subsequent to the one-time PDT treatment. In contrast, untreated tumors (outside the circles) continued to grow. The ^1^O_2_ measurement was correlated to the tumor reduction rate, as shown in Figure 5c. A linear fit to the data points demonstrates the positive correlation between ^1^O_2_ measurement with tumor reduction rate. The R^2^ value for the fit is 0.66, demonstrating correlation between the singlet oxygen value measured by the CS spectrometer and the PDT treatment efficacy. The high SNR measurement of ^1^O_2_ and its positive correlation with tumor reduction demonstrates the value of the CS-based luminescence detection technology in monitoring and optimizing PDT treatment outcome.

**Figure 6:**
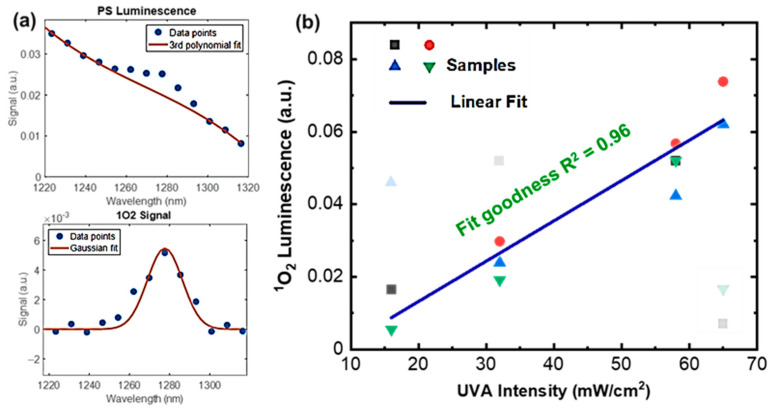
^1^O_2_ measurements during UVA irradiation on mouse skin in vivo. (**a**) Recorded spectra for PS (**top**) and ^1^O_2_ (**bottom**) for a living mouse (shown in **b**); (**b**) linear growth of measured ^1^O_2_ luminescence at different UVA (405 nm) light intensity levels. Outlier data points that are known due to experimental artifacts (e.g., mouse motion) were shaded and excluded from the fitting.

In a separate experiment, ^1^O_2_ measurements using the CS spectrometer were taken during an in vivo study of eight mice irradiated with UV light covering the wavelength range from 320 nm to 405 nm. The goal of the study was to measure ^1^O_2_ luminescence using the CS spectrometer and correlate it with the intensity of the UV source. Figure 6a shows the luminescence spectra from PS (originating from natural fluorophores in the mouse skin) and ^1^O_2_, both produced by UV irradiation of the skin. Again, to the best of our knowledge, this is the first high-resolution PS and ^1^O_2_ spectral measurement from a living mouse under UV radiation. Figure 6b shows the linear growth of ^1^O_2_ with the UVA intensity, and the R^2^ value of the linear fit to the measurement data is 0.96. This result demonstrates the sufficient measurement sensitivity of the dosimeter and its capability to quantify ^1^O_2_ in vivo skin irradiation.

**Figure 7:**
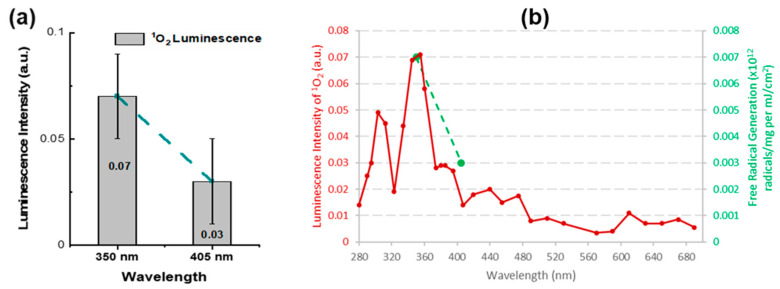
Wavelength dependence of ^1^O_2_ production measured using PSI CS-based dosimeter (**a**) and reactive oxygen species as a function of irradiation wavelength (measured ex vivo using electron spin resonance by Zastrow et al. [53]. Red line is a reproduction based on Zastrow data. Green dashed line indicating dosimeter measurements at 350 nm and 405 nm in (**b**) is consistent with PSI ^1^O_2_ dosimeter in vivo data shown in (**a**).

Figure 7 represents a measurement of the ^1^O_2_ produced by two UV wavelengths, i.e., 350 nm and 405 nm. This finding is consistent with the action spectrum of reactive oxygen species measured ex vivo using electron spin resonance (ESR) over a wide spectral range [53]. These results suggest that noninvasive ^1^O_2_ detection may be a good surrogate to measure ROS in vivo. Because ROS produced by UV irradiation are responsible for DNA mutations and skin cancer, the ^1^O_2_ detection may provide a valuable new tool for studies of UVA skin damage mechanisms.

**Figure 8:**
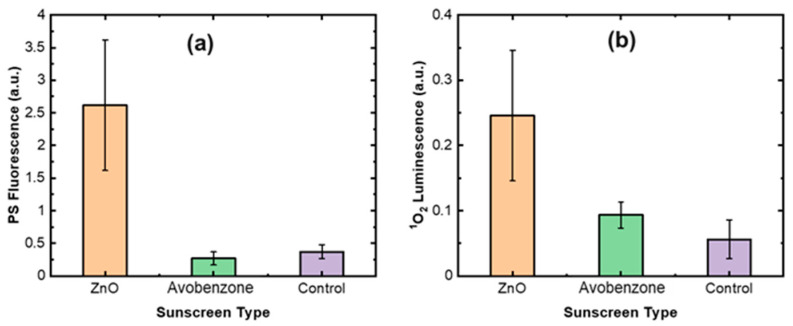
Comparison of (**a**) UVA-generated PS fluorescence and (**b**) ^1^O_2_ luminescence from in vivo mouse skin for two common sunscreen formulations.

Figure 8 shows the measured PS and ^1^O_2_ produced by UV on mouse skin when treated with commercial sunscreen products with either ZnO or avobenzone as the active ingredient. We observe that skin treated with the ZnO formulation produced up to five times as much PS and ^1^O_2_ luminescence as bare skin. This agrees with a previous study using a PMT-based monitor for singlet oxygen [54]. While at present it is unknown whether the ^1^O_2_ is produced only at the surface of the skin or in depth, it is clear that UV irradiated ZnO sunscreen produces much more ^1^O_2_ than does native skin. These preliminary data indicate that the CS-based ^1^O_2_ luminescence detection technology may be a valuable tool for studying the mechanisms of skin protection by sunscreen products, which in turn will support the development of new sunscreen formulations for UV protection, particularly in the longer wavelength UVA spectral region.

## 4. Conclusions

In this work, we demonstrate that the CS-based luminescence detection technique has the potential to provide an SNR and spectral resolution sufficient for non-invasive measurement of ^1^O_2_ produced during in vivo PDT treatment and UV skin irradiation. The positive correlations of ^1^O_2_ measurement with PDT treatment outcome (e.g., tumor reduction rate) and UV skin irradiation intensity demonstrate the value of this critical data to guide PDT dose optimization and to help better understand the UV skin damage mechanism and thus the development of improved sunscreen products. This simple, robust, and low-cost singlet oxygen sensor may become a valuable tool in these fields.

## Data Availability

Data supporting reported results are considered proprietary to Physical Sciences Inc. and cannot be released without signing a confidentiality agreement.
